# Non‐steroidal anti‐inflammatory drugs and risk of kidney cancer: A Swedish nationwide cohort study in the general and high‐use populations

**DOI:** 10.1111/joim.20079

**Published:** 2025-03-10

**Authors:** Hjalmar Wadström, Johan Askling, Rolf Gedeborg, Nils Feltelius, Karin Hellgren

**Affiliations:** ^1^ Clinical Epidemiology Division Department of Medicine Solna Karolinska Institutet Stockholm Sweden; ^2^ Medical Unit of Clinical Pharmacology Karolinska University Hospital Stockholm Sweden; ^3^ Rheumatology, Theme Inflammation and Ageing Karolinska University Hospital Stockholm Sweden; ^4^ Department of Surgical Sciences Uppsala University Uppsala Sweden; ^5^ Department of Public Health and Caring Sciences Uppsala University Uppsala Sweden; ^6^ Academic Specialist Center Stockholm Health Services Stockholm Sweden

**Keywords:** kidney cancer, mortality, NSAID, rheumatic disease

## Abstract

**Background:**

Data on the association between non‐steroidal anti‐inflammatory drugs (NSAIDs) and kidney cancer (KC) are conflicting. This study aimed to evaluate this association in the general population and in patients with extensive NSAID use: rheumatoid arthritis (RA) and spondyloarthritis (SpA).

**Methods:**

We conducted a nationwide register‐based cohort study of the Swedish general population and among patients with RA or SpA, among whom NSAID use was around five times higher. In each of these cohorts, we assessed the incidence of KC 2010 through 2021 by NSAID exposure as defined by repeated prescriptions. We also evaluated KC mortality in individuals treated (vs. not) with NSAIDs, taking the cancer stage into account. Adjusted hazard ratios (HRs) were calculated through Cox regression, taking age, sex, educational level, comorbidities and family history of KC into account.

**Results:**

Based on 751 incident cases of KC among 393,709 individuals in the general population (33% NSAID‐exposed), the HR for NSAID‐exposure was 1.32 (95% confidence interval [CI] 1.13–1.54), with the highest HRs during the first year of follow‐up (HR thereafter 1.20). The corresponding cancer stage‐adjusted HR for mortality from KC with NSAID‐exposure was 1.26 (95%CI 0.87–1.82). In RA and SpA, the HRs for KC incidence with NSAID exposure were 0.83 (95%CI 0.58–1.18) and 1.60 (95%CI 0.78–3.29), respectively.

**Conclusions:**

We found up to a 30% increase in the overall incidence and mortality from KC with NSAID in the general population. This association was attenuated beyond the first year of follow‐up and inconsistent in populations with much higher NSAID use.

## Graphical abstract

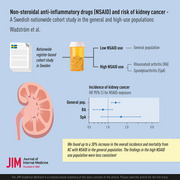

**Abbreviations**
AS: ankylosing spondylitis
ATC code: The Anatomical Therapeutic Chemical code
b/ts‐DMARDs: biologic or targeted synthetic disease–modifying antirheumatic drugs
csDMARD: conventional synthetic disease–modifying antirheumatic drugs
DDD: defined daily dose
HR: hazard ratio
ICD 10: International Statistical Classification of Diseases and Related Health Problems, 10
KC: kidney cancer
NCR: National Cancer Register
NPR: National Patient Register
NSAID: non‐steroid anti‐inflammatory drug
PDR: Prescribed Drug Register
RA: rheumatoid arthritis
RCC: renal cell carcinoma
SNOMED: Systematized Nomenclature of Medicine
SpA: spondyloarthritis
TNM: tumour, lymph node, metastasis

## Introduction

Due to their potent anti‐inflammatory properties, non‐steroidal anti‐inflammatory drugs (NSAIDs) are among the most widely used drugs worldwide [1]. NSAIDs, aspirin in particular, may reduce the risk of certain malignancies such as colorectal cancer [2]. Conversely, the potential association between NSAID use and kidney cancer (KC) is an open safety signal with contradictory results from previous studies [3, 4, 5, 6]. Some studies report an increased risk of the most common type of KC that is, renal cell carcinoma (RCC) with NSAIDs [3], at least in women [4]. In other studies, no such association has been found [5, 6].

This safety signal is of particular concern for patients with rheumatic diseases and consequent long‐term use of NSAIDs at higher doses, such as rheumatoid arthritis (RA) and spondylarthritis (SpA), including ankylosing spondylitis (AS). Thus, RA and SpA may serve as target populations in which any cancer risks with NSAIDs would be more easily detected (because of the higher doses and longer duration of exposure).

As for the underlying risk of KC in chronic inflammatory arthritis, some studies have signalled increased risks in patients with AS [7, 8, 9, 10], whereas others have not [11, 12]. For RA, a study by Chen et al. from Taiwan reported an increased risk for RCC, a finding that was not replicated in another study based on the same data [13, 14].

The primary aim of this study was, therefore, to estimate the association between NSAID use and the risk of KC in the general population and in patients with RA or AS/SpA and whether these associations differ by age, sex, duration of NSAID use and cumulative dose of NSAIDs. Moreover, an increased detection of early‐stage tumours may introduce detection bias in studies examining the risk of KC in populations at close(r) medical contact. A secondary aim was, therefore, to assess the stage at diagnosis and to evaluate KC mortality by NSAID exposure, taking the KC stage into account.

## Methods

### Study design, setting and data sources

We performed a nationwide observational cohort study using prospectively collected individual‐level, clinical‐ and register data.

In Sweden, healthcare has a high degree of public funding and coverage is universal. Prescribed drugs for continuous use are typically dispensed for up to a 3‐month supply. Patients with RA and SpA are typically cared for by hospital‐based rheumatologists. For this study, we used data from the National Patient Register (NPR) [15], the Prescribed Drug Register (PDR) [16], the National Cancer Register (NCR) [17], the Cause of death register [18], the Multi‐generation register [19] and the Swedish Rheumatology Quality (SRQ) register [20]. The linkage between registers was made possible by the unique personal identification number assigned to all residents in Sweden. The data sources and the information collected from them are described in Table S1. The register linkages used for this project have been described in detail elsewhere [21].

### Study population

For the RA and the SpA cohort, we identified all individuals above 18 years of age with at least two visits in the outpatient part of NPR between the 1 January 2006 and the 31 December 2021 with a diagnosis of RA (International Statistical Classification of Diseases and Related Health Problems, 10 [ICD 10]‐codes M05.9 or M06.0), or SpA (ICD 10‐codes M45.9, M46.8 or M46.9), respectively. At least one of the visits had to have been at a rheumatology or internal medicine department. The date of the second visit served as our date of inclusion. The RA and SpA diagnoses in NPR have high validity, with positive predictive values of about 80%–90% when validated against classification criteria [22, 23]. A flow chart illustrating the cohort establishment, exposure classification and outcome assessment periods is presented in Fig. S1.

Originally, each individual with RA or SpA was matched on age (at inclusion), sex and residential area to five general population individuals from the Total Population register. As the main exposure of interest for this study was NSAID and not the inflammatory arthritis condition itself, this existing matching was broken, and the general population comparators cohort were (just as the RA and SpA cohorts) categorized according to time‐varying treatment with (vs. not) NSAID.

### NSAID exposure

Information on NSAID was collected from the PDR using filled prescriptions of the Anatomical Therapeutic Chemical code (ATC code) M01A (except glucosamine M01AX05) or acetylsalicylic acid between the 1 January 2006 and the 31 December 2021. For detailed ATC codes, see Table S2. Exposure to NSAID was defined in two ways, described in more detail in the supplement (Supporting Information notes):

**Ever regular use**: at least two filled NSAID prescriptions within a 6‐month period. For this definition, individuals in each of the three cohorts were categorized as unexposed up until their second (if any) dispensation of any NSAID drug and as exposed thereafter. One individual could thus contribute person–time and events both as unexposed and (following fulfilment of the exposure definition) as exposed.

**Cumulative use**: as treatment with NSAID may be of a time‐varying nature, we constructed models to define cumulative use. For each dispensed NSAID prescription, we estimated the days of treatment as the amount of dispensed drug divided by the defined daily dose (DDD) of each prescribed NSAID, as defined by the WHO [24]. Assuming that all dispensed drugs were also used (returned unused medicines were subtracted from the accumulated dispensed prescriptions), the cumulative use was categorized as <1, 1 to <4 and ≥4 years. Thus, irrespective of which NSAID was dispensed or whether the NSAID use was continuous or not, each individual's NSAID exposure accrued with each unique dispensation during the study period.

### Outcome

We identified incident KC as an ICD 10‐code of C64 in NCR (excluding cancer in situ) and/or the Cause of death register (main or contributory diagnosis). To avoid a previous cancer affecting the prescription and use of NSAID, or the risk of developing or detecting a KC, all individuals with a history of a previous invasive cancer (ICD 10 C00‐C97 in NCR, excluding non‐melanoma skin cancer) at the start of follow‐up were excluded. The primary outcome was defined as a first invasive KC during follow‐up. Among individuals who developed the primary outcome, a secondary outcome was defined as death with KC as the underlying cause.

### Covariates

Status for all covariates was assessed at entry into the three cohorts (general population, RA and SpA) and treated as time‐fixed but was updated at any change of exposure‐status that resulted in transfer to another cohort.

Using the linked registry data, we extracted information on sex, year of start of follow‐up, educational level and history of KC in siblings. We also collected (ever/never) information on comorbid conditions at cohort entry, including congestive heart failure, diabetes mellitus, hypertension, chronic obstructive pulmonary disease, ischaemic heart disease, chronic kidney failure, stroke, venous thromboembolism, urolithiasis and hospitalized infections (within the last 5 years before the start of follow‐up) subcategorized into infections related to the urinary system and other infections. We additionally extracted dispensations of low‐dose acetylsalicylic acid and paracetamol. For RA and SpA, we also extracted information on joint surgery (within the last 5 years), concomitant antirheumatic treatments at the time of cohort entry categorized as biologic or targeted synthetic antirheumatic disease modifying drugs, conventional synthetic DMARDs (csDMARDs) and oral glucocorticoid treatment. Disease duration was defined as the time elapsed between the first registered RA or SpA diagnosis in NPR and cohort entry. ICD 10 and ATC codes used to define our covariates are listed in Table S3.

### Statistical analyses

In each of the three cohorts, we estimated crude and age‐standardized incidences of KC in relation to NSAID use (yes/no, time‐dependently) and applied Cox proportional hazards regression models to calculate hazard ratios (HRs) with 95% confidence intervals (CIs) using attained age as timescale. To investigate any non‐proportionality of HRs we stratified the analyses on attained age. To further investigate any non‐proportionality of HRs and to test the robustness of the results, an alternative timescale (time since the start of follow‐up) was also applied. In addition to analysing each cohort separately (within‐cohort analyses), we combined the three cohorts and estimated the risk of KC with NSAID exposure using unexposed in the general population as a reference (between‐cohort analyses). To account for the dependence between the RA and SpA cases and the general population introduced by the original matching, the between‐cohort analyses were performed using a cluster‐robust sandwich variance estimator.

The start of follow‐up among NSAID‐exposed was 1 January 2010, inclusion date, or the date of a second dispensed NSAID within a 6‐month period counting from 2006, whichever occurred later. The start of follow‐up among the NSAID‐unexposed was 1 January 2010, or the inclusion date, whichever occurred later. 1 January 2010, was chosen as the earliest start of follow to minimize the risk of misclassification stemming from left truncation of NSAID exposure data before the inception of PDR in 2005. Since the exposure was defined as NSAID dispensation beginning the earliest on 1 January 2006, NSAID exposure could be accrued before the start of follow‐up. For the RA and SpA cohorts, NSAID prescriptions dispensed during the (immortal) time before their index dates (second RA or SpA diagnosis) were excluded from the main analyses. We also performed an analysis using an alternative exposure definition where these dispensed prescriptions were included.

End of follow‐up was defined as the first invasive KC, other invasive cancer, date of any emigration, death, or end of the study period (31 December 2021), whichever came first.

We calculated crude incidence rates by exposure and cohort and applied two incrementally adjusted Cox models to calculate HRs for NSAID exposure: HRa adjusted for sex and calendar year and implicitly adjusted for age (as underlying timescale), and HRb additionally adjusted for comorbidities (Table S3), educational level and family history. For the within‐cohort analyses of the RA and SpA cohorts, HRb we also adjusted for concomitant antirheumatic treatments and disease duration. We performed analyses stratified by sex and age at entry. We also performed analyses stratified per cumulative NSAID use (<1, 1–4, >4 years) and by time since the start of the fulfillment of NSAID exposure (0–6, 6–12, >12 months) and introduced a 1‐year lagged start of follow‐up to explore reverse causality or any increased detection of KC in conjunction with the start of treatment with NSAID. We only presented HRs if the number of events in each cohort was ≥5.

To measure the robustness of our findings to potential unmeasured or uncontrolled confounding, we additionally calculated *E*‐values for the primary outcome as described by VanderWeele and Ding [25].

For our secondary aim, we assessed mortality from KC (among subjects diagnosed with KC) in each cohort by NSAID exposure status at the time of the KC diagnosis using two Cox models: HRa adjusted for calendar year and sex, and HRb adjusted for calendar year, sex and tumour, lymph node, metastasis (TNM) cancer stage at diagnosis. There was a low degree of missingness for TNM cancer stage at diagnosis (approximately 5%) and educational level (<1%). Apart from that, missingness was negligible. All analyses were performed on complete case data.

### Sensitivity analyses

Because we did not have access to data on smoking for the general population, we assessed the impact of smoking in the RA and SpA cohorts. These analyses included the subset of RA and SpA patients registered in the SRQ Register with smoking data. Follow‐up started from the visit when smoking status was first recorded in SRQ, that is, 2012, until 2021.

Although NCR coverage is high, some cancers are known to be underreported and are only identified in the Cause of death register [26]. In a sensitivity analysis, we excluded such KCs.

Finally, we performed an analysis restricted to clear cell carcinoma, the most common subtype of KC.

Analyses were performed using SAS, version 9.4.

### Ethical consideration

This study was approved by the Swedish Ethics Review Agency (2015‐1844‐31/2) and performed in accordance with the Declaration of Helsinki.

## Results

### Baseline characteristics

In the general population cohort of 393,709 individuals, 33% contributed NSAID‐exposure. NSAID‐exposure was associated with age (higher), sex (females) and educational level (lower), use of paracetamol and low dose ASA at start of follow‐up (more common), as well as comorbidities (higher prevalences), Table 1.

**Table 1:** Characteristics individuals in three cohorts (general population, rheumatoid arthritis, spondyloarthritis) by NSAID exposure.

	General population		Rheumatoid arthritis		Spondyloarthritis	
NSAID exposure definition^a^	Unexposed	Exposed	Unexposed	Exposed	Unexposed	Exposed
***N*. individuals**	307,382	131,686	49,073	32,626	16,513	13,601
**Mean age, at start of follow‐up, years (IQR)**	57 (43–68)	62 (51–71)	64 (52–73)	62 (51–70)	42 (32–54)	45 (35–55)
**Female, percentage**	64	70	71	75	45	46
**Year of start of follow‐up median (IQR)**	2011 (2010–2015)	2013 (2010–2017)	2012 (2010–2016)	2011 (2010–2015)	2014 (2011–2018)	2013 (2010–2017)
**Education level at start of follow‐up, percentage**						
<12 years	48	56	60	60	35	38
≥12 years	50	43	39	39	64	62
**Sibling with kidney cancer, percentage**	0.1	0.1	0.1	0.1	0.0	0.0
**Median disease duration years (IQR)**	–	–	1 (0–5)	4 (2–8)	1 (0–3)	3 (1–7)
**Treatment at start of follow‐up, percentage**	–	–				
Low dose acetylsalicylic acid	11	16	18	14	5	5
Paracetamol	16	47	52	62	43	52
**Antirheumatic treatment at start of follow‐up, percentage**						
b/ts‐DMARD	–	–	10	24	16	25
cs‐DMARD	–	–	66	89	28	40
Oral glucocorticoids	–	–	59	64	29	32
**Comorbidity (ever) at start of follow‐up, percentage**						
Congestive heart failure	2	2	5	3	1	1
Diabetes	2	3	4	4	1	2
Hypertension	11	19	23	20	9	10
Chronic obstructive pulmonary disease	2	2	4	4	1	1
Ischaemic heart disease	5	7	10	7	3	3
Kidney failure	0	0	0	0	0	0
Stroke	3	4	5	3	1	1
Venous thromboembolism	1	2	3	2	1	1
Urolithiasis	2	4	3	3	3	4
Hospitalized Infections within the last 5 years	2	3	6	6	4	4
Hospitalized urinary infections within the last 5 years	1	1	1	1	1	1
Joint surgery within the last 5 years	1	2	5	5	1	1

Abbreviations: b/ts‐DMARD, biologic or targeted synthetic disease modifying anti rheumatic drug; cs‐DMARD, conventional synthetic DMARD; IQR, interquartile range; NSAID, non‐steroidal anti‐inflammatory drug.

^a^NSAID exposure definition: Ever regular use defined as at least two filled NSAID prescriptions within a six‐month period. Individuals in each of the three cohorts were categorised as unexposed up until their second (if any) dispensation of any NSAID drug, and as exposed thereafter. One individual could thus contribute person‐time and events both as unexposed and (following fulfilment of the exposure definition) as exposed.

In the RA cohort of 62,944 patients, 32,626 (52%) were exposed to NSAID. NSAID‐exposure was associated with age (younger), sex (females), RA disease duration (longer) and use of paracetamol, csDMARDs and b‐/ts‐DMARDs at the start of follow‐up (more).

In the SpA cohort of 20,111 patients, 13,601 (68%) were exposed to NSAID. Because many AS/SpA patients started follow‐up as unexposed only to later fulfil our definition of NSAID exposure, the mean follow‐up was shorter among NSAID unexposed than among the exposed. NSAID‐exposure was associated with age (older), disease duration (longer) and use of paracetamol, csDMARDs and b‐/ts‐DMARDs at the start of follow‐up (more), Table 1.

### Relative risk of KC in the general population

**Fig. 1:**
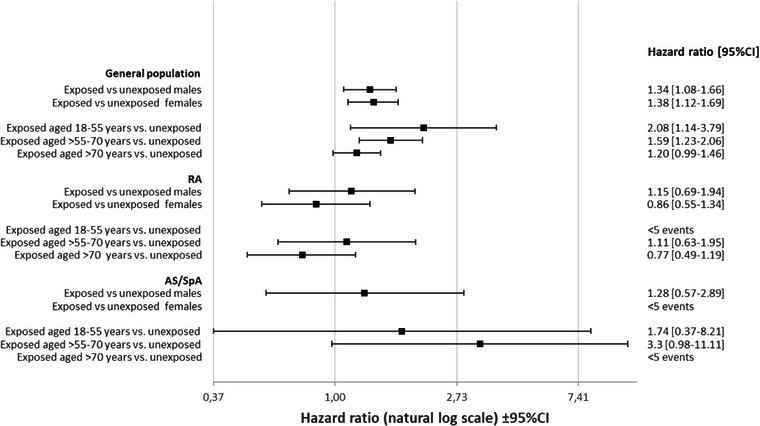
Risk of kidney cancer (KC) stratified by sex and age categories. Adjusted for sex and calendar year and inherently adjusted for age (timescale attained age). AS, ankylosing spondylitis; CI, confidence interval; RA, rheumatoid arthritis; SpA, spondylarthritis.

**Fig. 2:**
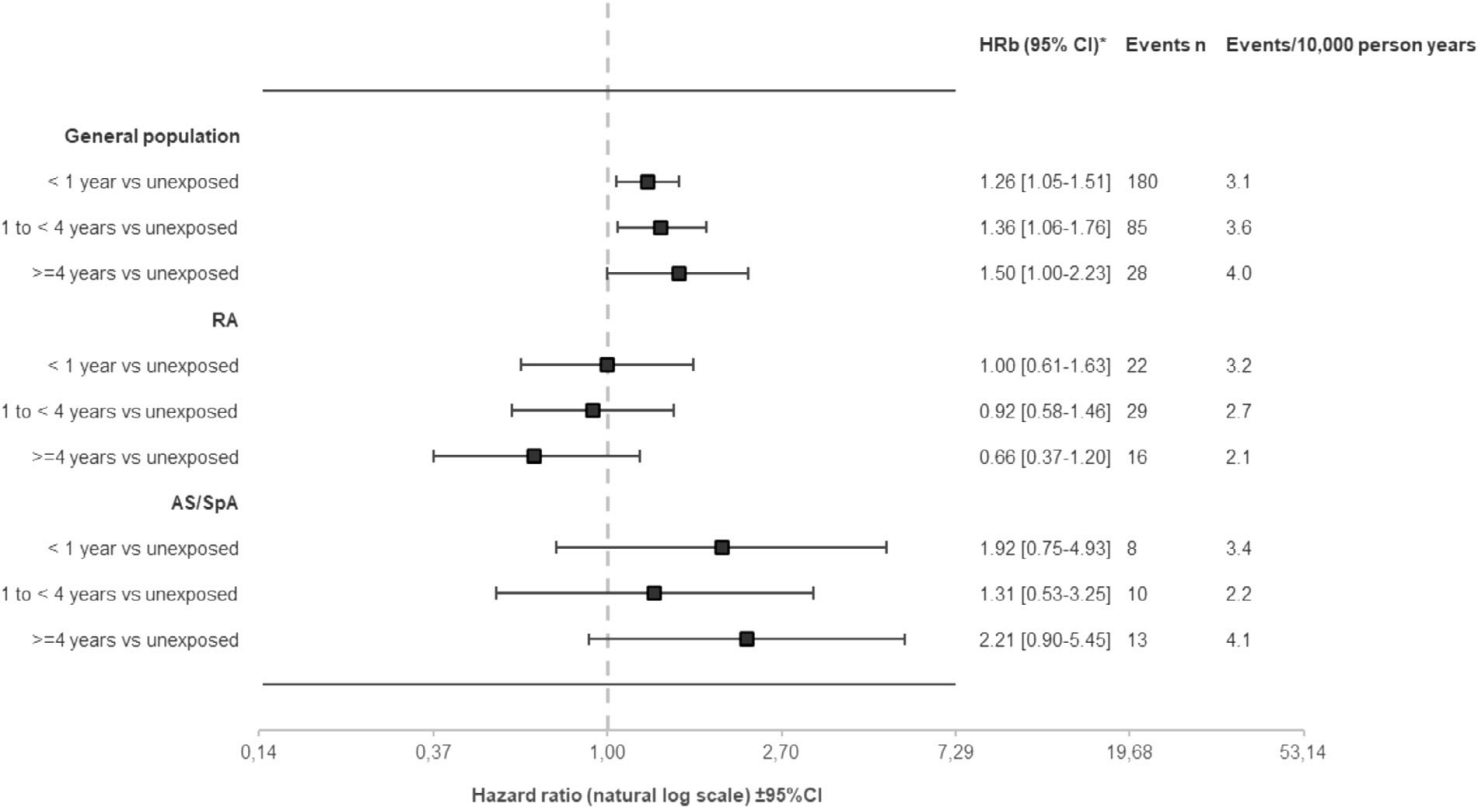
Risk of kidney cancer (KC) by cumulative years of non‐steroidal anti‐inflammatory drug (NSAID) exposure (vs. unexposed). Fully adjusted hazard ratios, events and crude incidences. *Adjusted for calendar year, sex, education, use of paracetamol, use of low dose ASA, KC in a full sibling, comorbidities (CHF, diabetes, hypertension, COPD, ischaemic heart disease, renal failure, stroke, venous thromboembolism, hospitalized infections total and hospitalized infections, urogenital infections, urolithiasis). Rheumatoid arthritis (RA) and ankylosing spondylitis (AS) analyses additionally adjusted for rheumatic disease duration, use of glucocorticoids, csDMARDs and bDMARDs at start of follow‐up. Attained age as the underlying timescale. CI, confidence interval; SpA, spondylarthritis.

During a mean follow‐up of 7 years (irrespective of exposure), we identified 293 incident KC among NSAID exposed (3.3 per 10,000 person‐years) and 458 incident KC among unexposed (2.1 per 10,000 person‐years), resulting in an HRb for NSAID of 1.32 (95%CI 1.13–1.54), Table 2. The results did not differ by sex, Fig. 1. When stratifying on three different age bands, we observed a weak trend towards decreasing risk with NSAIDs with increasing age, however with largely overlapping CIs, Fig. 1. Within‐cohort analyses stratified by cumulative NSAID use (with unexposed to NSAIDs as referent) showed a weak and uncertain trend towards increasing risk with increasing cumulative exposure, with an HRb of 1.26 (95%CI 1.05–1.51) for <1‐year exposure, and an HRb of 1.50 (95%CI 1.00–2.23) for ≥4 years exposure, Fig. 2. Applying an alternative categorization of cumulative NSAID exposure (<1, 1 to <4, 4 to<7, 7 to<10 and ≥10 years) for KC risk in the general population resulted in a largely similar pattern, although precision was limited beyond 7 years of use, Table S4. Stratifying by time since start of NSAID exposure (vs. not) revealed a markedly increased risk in the 0–6 months following fulfilment of our NSAID exposure definition; HRb = 2.80 (95%CI 1.60–4.89) and during the 6–12 months thereafter (HRb = 2.97; 95%CI 1.76–5.02), which was attenuated thereafter (HRb = 1.24; 95%CI 1.04–1.48), Table 3.

**Table 2:** Crude incidence rates and hazard ratios (HRs) of kidney cancer with NSAID exposure (ever regular use), in three cohorts (general population, rheumatoid arthritis, and spondyloarthritis.

					Within‐cohort comparison		Between‐cohort comparison^c^	
	NSAID exposure	Individuals	Events of Kidney cancer	Crude incidence rate per 10,000	HRa (95% CI)^a^	HRb (95% CI)^b^	HRa (95% CI)^a^	HRb (95% CI)^b^
General population	No	307,382	458	2.1	1 (reference)	1 (reference)	1 (reference)	1 (reference)
	Yes	131,686	293	3.3	1.35 (1.17–1.57)	1.32 (1.13–1.54)	1.35 (1.16–1.56)	1.29 (1.10–1.50)
Rheumatoid arthritis	No	49,073	77	3.4	1 (reference)	1 (reference)	1.26 (0.99–1.60)	1.15 (0.90–1.48)
	Yes	32,626	64	2.6	0.89 (0.63–1.24)	0.83 (0.58–1.18)	1.10 (0.84–1.43)	1.06 (0.81–1.39)
Spondyloarthritis	No	16,513	12	2.3	1 (reference)	1 (reference)	1.79 (1.01–3.19)	1.68 (0.94–2.99)
	Yes	13,601	30	3.0	1.19 (0.61–2.34)	1.60 (0.78–3.29)	2.33 (1.60–3.39)	2.24 (1.53–3.28)

Abbreviations: CI, confidence interval; NSAID, non‐steroidal anti‐inflammatory drug.

^a^ HRa Adjusted for calendar year, sex with attained age as the underlying timescale.

^b^ HRb Adjusted for calendar year, sex, education, use of paracetamol, use of low‐dose ASA, KC in a full sibling, comorbidities (CHF, diabetes, hypertension, COPD, ischaemic heart disease, renal failure, stroke, venous thromboembolism, hospitalized infections total and hospitalized infections, urogenital infections, urolithiasis). The within‐cohort analyses for the RA and SpA cohorts were additionally adjusted for rheumatic disease duration, use of glucocorticoids, csDMARDs and bDMARDs at start of follow‐up. Attained age as the underlying timescale.

^c^ The different cohorts were pooled together in the between‐cohort analyses. To handle the dependence arising from matching they were performed using a cluster‐robust sandwich variance estimator.

**Table 3:** Hazard ratios (HR) of kidney cancer in three cohorts (general population, rheumatoid arthritis and spondyloarthritis) by time since fulfilment of NSAID exposure.

	HRb^a^ (95% CI)		
Time since NSAID exposure fulfilment	General population	Rheumatoid arthritis	Spondyloarthritis
Unexposed	Reference	Reference	Reference
0–6 months	2.80 (1.60–4.89)	NA (<5 events)	NA (<5 events)
>6–12 months	2.97 (1.76–5.02)	2.02 (0.85–4.79)	NA (<5 events)
>12 months	1.24 (1.04–1.48)	0.76 (0.52–1.12)	1.59 (0.76–3.33)

Abbreviations: CI, confidence interval; NSAID, non‐steroidal anti‐inflammatory drug.

^a^ HRb = Adjusted for calendar year, sex, education, use of paracetamol, use of low‐dose ASA, KC in a full sibling, comorbidities (CHF, diabetes, Hypertension, COPD, ischaemic heart disease, renal failure, stroke, venous thromboembolism, hospitalized infections total and hospitalized infections, urogenital infections, urolithiasis). RA and AS analyses additionally adjusted for rheumatic disease duration, use of glucocorticoids, csDMARDs and bDMARDs at start of follow‐up. Attained age as the underlying time scale. NA = not applicable due to few events.

### Relative risk of KC in RA

During a mean follow‐up of 8 (NSAID exposed) and 5 (unexposed) years, 64 incident KC occurred among NSAID‐exposed RA (2.6 per 10,000 person‐years) and 77 among unexposed RA (3.4 per 10,000 person‐years), HRb 0.83 (95%CI 0.58–1.18), Table 2. The result did not substantially change when stratifying by age categories or sex (Fig. 1). The risk of KC neither increased with cumulative NSAID use nor with time since the start of NSAID exposure fulfilment (Fig. 2 and Table 3).

In the between‐cohort analyses, comparing the RA cohort to the general population cohort, with unexposed to NSAID in the general population as the referent, there was no sign of an increased risk of KC in RA per se, Table 2.

### Relative risk of KC in SpA

During a mean follow‐up of 7 (NSAID exposed) and 3 (unexposed) years, 30 incident KC occurred among NSAID‐exposed SpA (3.0 per 10,000 person‐years) and 12 among unexposed SpA (2.3 per 10,000 person‐years), HRb = 1.60 (95%CI 0.78–3.29), Table 2. Analyses stratified by age and sex were hampered by low precision, Fig. 1. When stratified by cumulative NSAID use, there was no clear trend of increasing risk with cumulative use, and the HR estimates had poor precision, Fig. 2. There were too few events to calculate HRs stratified by time since the start of the fulfilment of NSAID exposure, Table 3.

In between‐cohort analyses comparing the SpA cohort to the general population cohort with unexposed to NSAID in the general population as the referent, SpA seemed to be associated with increased risk whether unexposed, HRa = 1.79 (1.01–3.19) or exposed, HRa = 2.33 (1.60–3.39) to NSAIDs, although precision was low, Table 2.

### Additional analyses

For both the RA and the SpA cohort, analyses using an alternative exposure definition where prescriptions dispensed before the date of inclusion were included yielded results close to the main analyses, Table S5.

For all three cohorts (general population, RA and SpA), using time since the start of follow‐up as an alternative timescale (instead of attained age) in the within‐cohort analyses resulted in similar results (Table S6). Likewise, we observed largely similar results when we excluded the first year of follow‐up from the main analyses (Table S7).

### Kidney cancer stage and mortality

Apart from relatively more stage IV tumours in RA patients unexposed to NSAID compared to exposed RA patients, the TNM cancer stage at diagnosis was similar between NSAID‐exposed and unexposed within the different cohorts, Table S8.

When we analysed mortality with KC as the underlying cause of death among KC cases in the general population (with NSAID‐unexposed in the general population as referent), we noted an association with higher mortality among NSAID‐exposed taking sex and calendar year into account, HRa = 1.63 (95%CI 1.14–2.32). Further adjustment for TNM cancer stage considerably attenuated this association, HRb = 1.26 (95%CI 0.87–1.82), Table S9.

Conversely, compared to NSAID‐unexposed in the general population (referent), the risk of death with KC as the cause of death was higher in RA patients unexposed to NSAID, HRb = 2.26 (95%CI 1.32–3.88) than in NSAID‐exposed RA patients, HRb = 1.00 (0.50–1.98).

For SpA, the analysis was hampered by the low number of events. The risk of death was increased for NSAID‐exposed SpA patients compared with unexposed in the general population (HRa, 1.89; 95%CI 0.88–4.04) based on eight deaths. Adjusting also for TNM cancer stage increased the HR further, HRb = 3.67 (95%CI 1.54–8.70), Table S9. As there were fewer than five cases of death with KC as the underlying cause in NSAID‐unexposed with SpA, we abstained from calculating HRs.

### Sensitivity analyses

In the subset of RA and SpA patients registered with smoking data in the SRQ Register 2012 and later (*n* = 51,246), we adjusted the base model (HRa) of NSAID‐exposed versus unexposed for smoking status (ever/never). This did not alter the relative risk for KC with NSAID exposure (Table S10).

Restricting the analysis to KC of clear cell carcinoma type did not yield materially different results (Table S11), nor did including only cases of KC registered in the NCR, that is, excluding all cases retrieved only from the Cause of death register (Table S12).

To examine potential residual confounding, we switched the main exposure from regular use of NSAID to regular use of paracetamol. This yielded an increased risk of KC in the general population in our base model HRa = 1.46 (1.26–1.70), which was attenuated in the fully adjusted model HRb = 1.23 (95%CI 1.03–1.50). This analysis was additionally adjusted for the use of NSAIDs instead of paracetamol at baseline.

The calculated *E*‐value suggests that the observed HR of 1.32 for the primary outcome in the general population would be completely explained by an unmeasured confounder that was associated with both the outcome and our NSAID exposure variable by a risk ratio (for each) of 1.97.

## Discussion

This study has several key findings. First, in the general population, exposure to NSAIDs was associated with a 30% increase in both the risk of and mortality from KC, which corresponds to an absolute risk increase of about one additional annual case for every 15,000 treated individuals. We found weak trends for an increased risk with cumulative use but also a weaker association beyond the first year after the fulfilment of our NSAID exposure definition. Second, in RA, where the proportion of NSAID exposure (and the volume of exposure) is higher than in the general population, there was no association between NSAID exposure and KC risk. Third, for SpA, we observed an increased risk of KC per se, and even if the point estimate for NSAID use was increased, precision was limited.

Our finding of a 30% increased risk of KC with NSAID use in the general population is compatible with some previous studies [3, 4, 27]. Bruinsma et al. reported an odds ratio of 1.32 for non‐aspirin NSAIDs in a large case–control study [4]. The risk was only increased among women in their study, whereas we did not find that the risk for KC with NSAID differed by sex. A register‐based study from Denmark reported an SIR for KC of 1.2 (1.0–1.5) in the general population defining the use of NSAID as only one prescription of NSAID in the Danish PDR and a significant trend of increasing risk with an increasing number of prescriptions [27]. Cho et al. used data from two large US cohorts and found a 50% increased risk of the most common subtype of KC, RCC with regular NSAID use [3]. Further, a meta‐analysis by Choueiri et al., including some of the above studies, reported a pooled relative risk for KC with non‐aspirin NSAID of 1.25 (95% CI: 1.06–1.46), with a higher risk with increasing exposure [28]. Although there were differences in both exposure (any use, regular use) and outcome definitions across included studies, there was no significant inter‐study heterogeneity.

Contrary to this, two US studies reported no association between use of NSAIDs and RCC (the most common subtype of KC) [5, 6]. The study by Karami et al. analysed the risk of RCC in both a case–control and cohort design and found no association with NSAID use in either study [5]. Liu et al. observed no overall association between RCC and NSAID use, although there was some evidence of an increased risk of RCC with frequent NSAID use among younger individuals [6].

It may be that differences in the definition of NSAID exposure explain some of the heterogeneity in results. Our register‐based definition of exposure was at least two dispensed NSAID prescriptions within 6 months and may have missed some true regular use but with less frequently dispensed prescriptions and overestimated true exposure if dispensed drugs were not consumed. By design, our exposure definition did not accommodate NSAID exposure earlier than 2006 and did not include over‐the‐counter use. By contrast, studies using questionnaire data [3–5, 29] may have been susceptible to recall bias as well as non‐response.

An increased risk of KC among NSAID‐exposed in the general population could be due to detection bias from more frequent healthcare contacts. In fact, data indicate that the majority of KC cases in Sweden are diagnosed during imaging performed for other reasons [30]. Among the minority of patients presenting with symptoms, flank pain is common. NSAIDs may, therefore, be prescribed to treat early symptoms of undiagnosed KC. Indeed, we found a higher risk of KC during the first year following NSAID exposure fulfilment, which is arguably too short a follow‐up for any causal effect by NSAIDs on KC occurrence and might suggest detection bias, reverse causation, or both. However, the HR remained elevated, albeit at a lower level (HRb = 1.24) after the first year, indicating that such bias can only partly explain the association. Further, our adjustment for comorbid conditions and medications should have captured some of the effects of frequent healthcare contacts but did not substantially change HRs. However, the fact that switching our exposure from regular use of NSAID in the general population to regular use of paracetamol resulted in an elevated HR of KC between that of NSAID and the null could indicate residual confounding by indication, although some studies have reported an association between paracetamol and KC risk [4, 28, 29]. Additionally, there was no sign of lower mortality in KC that had arisen in NSAID‐exposed as compared to unexposed, and within the different cohorts, TNM cancer stage at diagnosis was largely similar by NSAID‐exposure.

Even though NSAID use in NSAID‐exposed RA patients was severalfold higher than in NSAID‐exposed individuals in the general population (median dispensed DDDs during follow‐up of 2.5 years vs. 0.8 years), we found no association between NSAIDs and KC risk in patients with RA, rather the opposite. In RA, most NSAID use is presumably linked to rheumatic disease, whereas in the general population, the underlying indications probably reflect an array of acute or chronic pain‐related conditions. Thus, the discrepancies in HR between the general population and RA may reflect confounding by indication rather than effects of NSAIDs per se.

Although not the focus of this study, we noted an elevated risk for KC in SpA patients per se, regardless of NSAID use. This finding is in line with several [7, 8, 10] but not all previous studies [11, 12]. A recently published Mendelian randomization study found a positive association between both AS and SpA, respectively, and renal malignancies [31]. Kidney stones are also more common in AS/SpA and, although we adjusted for urolithiasis, there may still be some residual confounding [32]. A possible explanation (other than any true association with NSAIDs) could thus be detection bias due to the use of imaging and the intensity of healthcare contacts in SpA. Importantly, our results regarding NSAID use and KC risk in SpA should be interpreted with caution as the precision was limited.

NSAIDs may cause a wide spectrum of chronic and acute kidney damage [33] directly related to their pharmacological effect that is, inhibition of prostaglandin synthesis, affecting sodium homeostasis and blood pressure. Kidney damage may also arise from unrelated mechanisms, such as hypersensitivity reactions, including interstitial nephritis [34]. Whether such effects of NSAIDs would lead to an increased KC risk remains, however, unknown [35, 36].

Our study has several strengths, including our use of nationwide, population‐based prospective health registers with high coverage and validity. Our study is also one of the largest ones on the risk of KC with NSAID use to date. Cancer outcomes were ascertained independent of exposure, thereby minimizing the risk of recall or ascertainment bias, and exposure was determined using complete dispensation data rather than self‐reported. We found that the rates of KC in our general population comparator were the same as age‐ and sex‐standardized rates from the National Board of Health and Welfare, which indicates that the internal validity was good and that the observed observations are generalizable to individuals in Sweden with a similar age‐ and sex distribution as our general population cohorts. Assessing both the incidence and mortality of KC, and using data on TNM cancer stages, permitted assessment of potential detection bias. Moreover, we were able to adjust for several potential confounders, including both comorbid conditions of interest, family history of KC and in a subgroup analysis also smoking. We also evaluated the robustness of our results by applying an alternative timescale of the Cox regression model (i.e., time since the start of follow‐up instead of attained age) and found substantially similar results. Additionally, we conducted analyses in the RA and SpA cohorts incorporating an alternative exposure definition that included prescriptions dispensed prior to the date of inclusion, and these analyses also produced largely similar outcomes.

Our study also has limitations. Some exposure misclassification is to be expected due to the lack of access to data on the over‐the‐counter use of NSAIDs, as well as the left truncation of data due to the PDR, which started in 2005. Moreover, we could not verify whether dispensed prescriptions were actually used. All of this may have introduced some level of misclassification of NSAID exposure. We could not adjust for obesity, considered a moderate risk factor for RCC [37, 38]. Although there may not be a direct link between obesity and NSAID exposure, an indirect link may exist through pain due to osteoarthritis, lower back pain and other unspecific musculoskeletal disorders. Finally, there could also be some misclassification of a few non‐RCC renal cancers captured through the cause of death register. However, when we restricted the analysis to cases from the NCR, the results were largely the same.

**In conclusion,** this so far largest cohort study indicates a 30% increased risk of KC with use of NSAID in the general population, but with only weak and uncertain evidence for any increased risk with cumulative exposure, an attenuated risk beyond the first year, and with no increased risk with NSAID use in the RA population with higher doses and longer duration of NSAID exposure. We have also confirmed previous findings of an increased risk of KC per se in patients with AS/SpA. The causality of our finding of a 30% relative risk increase of KC with regular NSAID use remains uncertain. These results should be interpreted with caution in clinical decision‐making.

## Supporting information




**Table S1**: Description of the registers used.
**Table S2**: ATC codes used to identify NSAIDs.
**Figure S1**: Flow chart illustrating the cohort establishment, exposure classification, and outcome assessment periods.
**Supplementary notes**: NSAID Exposure Classification by Dispensed Prescriptions and DDDs.
**Table S3**: Baseline Covariate Definitions.
**Table S4**: Risk of KC in the general population by cumulative NSAID exposure, alternative categorization of cumulative NSAID exposure.
**Table S5**: Crude incidence rates and hazard ratios of kidney cancer with NSAID prescriptions dispensed before the index date (second diagnosis of rheumatoid arthritis (RA) or spondyloarthritis (SpA) included..
**Table S6**: Hazard ratios of kidney cancer with ever regular use of NSAID in three cohorts using follow‐up time as the time‐scale instead of attained age.
**Table S7**: Hazard ratios of kidney cancer with ever regular use of NSAID in three cohorts, excluding the first year of follow‐up.
**Table S8**: TNM cancer stage grouping at diagnosis of KC by regular use of NSAID.
**Table S9**: Crude incidence rates and hazard ratios of death due to kidney cancer by ever regular NSAID use.
**Table S10**: Crude incidence rates and hazard ratio of kidney cancer in a subset of patients with rheumatoid arthritis (RA) and spondyloarthritis (SpA) 2012‐2021 by ever regular NSAID use, and further adjusted on ever smoking.
**Table S11**: Crude incidence rate and hazard ratios of kidney cancer by ever regular use of NSAID restricted to kidney cancer subtype clear cell carcinoma..
**Table S12**: Crude incidence rate and hazard ratio of KC by ever regular NSAID use including only KC recorded in the National Cancer Register.
