# Supplementary material for: Non‐steroidal anti‐inflammatory drugs and risk of kidney cancer: A Swedish nationwide cohort study in the general and high‐use populations
Source: J Intern Med. 2025 Mar 10;297(5):505–17. doi: 10.1111/joim.20079 (PMC12032997; doi:10.1111/joim.20079)
Supplement: Supplementary file 1 — Table S1: Description of the registers used. Table S2: ATC codes used to identify NSAIDs. Figure S1: Flow chart illustrating the cohort establishment, exposure classification, and outcome assessment periods. Supplementary notes: NSAID Exposure Classification by Dispensed Prescriptions and DDDs. Table S3: Baseline Covariate Definitions. Table S4: Risk of KC in the general population by cumulative NSAID exposure, alternative categorization of cumulative NSAID exposure. Table S5: Crude incidence rates and hazard ratios of kidney cancer with NSAID prescriptions dispensed before the index date (second diagnosis of rheumatoid arthritis (RA) or spondyloarthritis (SpA) included.. Table S6: Hazard ratios of kidney cancer with ever regular use of NSAID in three cohorts using follow‐up time as the time‐scale instead of attained age. Table S7: Hazard ratios of kidney cancer with ever regular use of NSAID in three cohorts, excluding the first year of follow‐up. Table S8: TNM cancer stage grouping at diagnosis of KC by regular use of NSAID. Table S9: Crude incidence rates and hazard ratios of death due to kidney cancer by ever regular NSAID use. Table S10: Crude incidence rates and hazard ratio of kidney cancer in a subset of patients with rheumatoid arthritis (RA) and spondyloarthritis (SpA) 2012‐2021 by ever regular NSAID use, and further adjusted on ever smoking. Table S11: Crude incidence rate and hazard ratios of kidney cancer by ever regular use of NSAID restricted to kidney cancer subtype clear cell carcinoma.. Table S12: Crude incidence rate and hazard ratio of KC by ever regular NSAID use including only KC recorded in the National Cancer Register. [file JOIM-297-505-s001.docx]

**Non-steroidal anti-inflammatory drugs (NSAID) and risk of kidney cancer - A Swedish nationwide cohort study** **in the general and in high-use populations**

Hjalmar Wadström MD, PhD, Johan Askling MD, Professor, Rolf Gedeborg MD, Associate professor, Nils Feltelius MD, Associate professor, Karin Hellgren MD, PhD

Supplementary Tables and Figures

Table of Contents

[Supplementary table 1. Description of the registers used 2](#_Toc664099604)

[Supplementary table 2. ATC codes used to identify NSAIDs 3](#_Toc1905387223)

[Supplementary figure 1. Flow chart illustrating the cohort establishment, exposure classification, and outcome assessment periods. 4](#_Toc481832772)

[Supplementary notes. NSAID Exposure Classification by Dispensed Prescriptions and DDDs 5](#_Toc1437057194)

[Supplementary table 3. Baseline Covariate Definitions 6](#_Toc1670812602)

[Supplementary table 4. Events, crude incidence rates and hazard ratios (HR) of KC in the general population by cumulative years of NSAID exposure measured by DDDs. Alternative categorization of cumulative years of NSAID exposure. 9](#_Toc488149278)

[Supplementary table 5. Crude incidence rates and hazard ratios (HR) of kidney cancer with NSAID prescriptions dispensed before the index date (second diagnosis of rheumatoid arthritis (RA) or spondyloarthritis (SpA) included. 9](#_Toc1492902605)

[Supplementary table 6. Hazard ratios (HR) of kidney cancer with ever regular use of NSAID in three cohorts using follow-up time as the time-scale instead of attained age 9](#_Toc1174068154)

[Supplementary table 7. Hazard ratios (HR) of kidney cancer with ever regular use of NSAID in three cohorts, excluding the first year of follow-up 10](#_Toc139185399)

[Supplementary table 8. TNM cancer stage grouping at diagnosis of KC by regular use of NSAID 10](#_Toc682777504)

[Supplementary table 9. Crude incidence rates and hazard ratios (HR) of death due to kidney cancer by ever regular NSAID use 11](#_Toc379991280)

[Supplementary table 10. Crude incidence rates and hazard ratio (HR) of kidney cancer in a subset of patients with rheumatoid arthritis (RA) and spondyloarthritis (SpA) 2012-2021 by ever regular NSAID use, and further adjusted by ever smoking 11](#_Toc2028234364)

[Supplementary table 11. Crude incidence rate and hazard ratios (HR) of kidney cancer by ever regular use of NSAID restricted to kidney cancer subtype clear cell carcinoma. 12](#_Toc952260762)

[Supplementary table 12. Crude incidence rate and hazard ratios (HR) of KC by ever regular NSAID use including only of KC recorded in National Cancer Register 12](#_Toc1744097685)

## **Supplementary table 1.** Description of the registers used

| **Register** | **Description** |
| --- | --- |
| **The Swedish Rheumatology Quality register (SRQ)** | A nationwide longitudinal clinically integrated register operated by the Swedish Society for Rheumatology, started in 1996. Patients with RA and other rheumatologic diseases are registered in the SRQ and it covers 89 000 patients. At start of treatment and at follow-up visits, the treating rheumatologist enters details of the disease activity and start and stop dates of each anti-rheumatic drug. Estimations against national prescription statistics have indicated that around 80% of all Swedish patients with RA are followed in SRQ and that >90% of all patients with RA treated with bDMARD are included in SRQ. |
| **The Swedish National Patient Register (NPR)** | A national register maintained by the National Board of Health and Welfare. Hospital discharges from inpatient care and patients visits in non-primary outpatient care, have been registered, since 1964 and 2001 respectively. Diagnoses are coded according to the Swedish version of the International Classification of Disease (ICD). |
| **The Swedish Prescribed Drug Register (PDR)** | A national register maintained by the National Board of Health and Welfare established in July 2005 PDR contains information on all dispensed prescriptions at Swedish pharmacies including date and the Anatomical Therapeutic Chemical (ATC)-code indicating the drug(s) in question. |
| **The National Cancer Register (NCR)** | A national register started in 1958. Reporting to the NCR is manatoryd for the clinicians and the coverage is estimated to more than 95%; the register contains data on date of diagnosis and type of incident cancers according to ICD codes and Systematized Nomenclature of Medicine (SNOMED) codes. |
| **The Cause of Death Register** | The Cause of Death Register is a national register containing information on date and cause of death (underlying and contributory) for all deceased residents, including deaths among Swedish residents who died abroad. The register was started in 1952, and the data is considered complete since 1961. From that year and onward, cause of death is missing for less than 0.5% of deceased individuals. |
| **The Total Population Register** | A national register maintained by Swedish Tax agency that contains information such as home district, civil status and migration data |
| **The multi-generation register (MGR)** | A nationwide register recording first degree (parents,siblings and children relatives to Swedish residents since 1961. |
| **Longitudinal database for insurance and labor market-studies (LISA)** | A national register maintained by Statistics Sweden. It contains information about sick leave, parental leave and employment status in Sweden since 1990 |

## **Supplementary table 2.** ATC codes used to identify NSAIDs

| **Substance** | **ATC code** |
| --- | --- |
| Sulindac | M01AB02 |
| Diclofenac | M01AB05, M01AB55 |
| Ketorolac | M01AB15 |
| Aceklofenac | M01AB16 |
| Ibuprofen | M01AE01 |
| Naproxen | M01AE02, M01AE52 |
| Ketoprofen | M01AE03 |
| Celecoxib | M01AH01 |
| Etoricoxib | M01AH05 |
| Lumiracoxib | M01AH06 |
| Nabumeton | M01AX01 |
| Dexibuprofen | M01AE14 |
| Indometacin | M01AB01 |
| Piroxicam | M01AC01 |
| Tenoxicam | M01AC02 |
| Lornoxikam | M01AC05 |
| Meloxicam | M01AC06 |
| Acetylsalicylic acid | N02BA01, N02BA51 |

##

## **Supplementary figure 1.** Flow chart illustrating the cohort establishment, exposure classification, and outcome assessment periods.


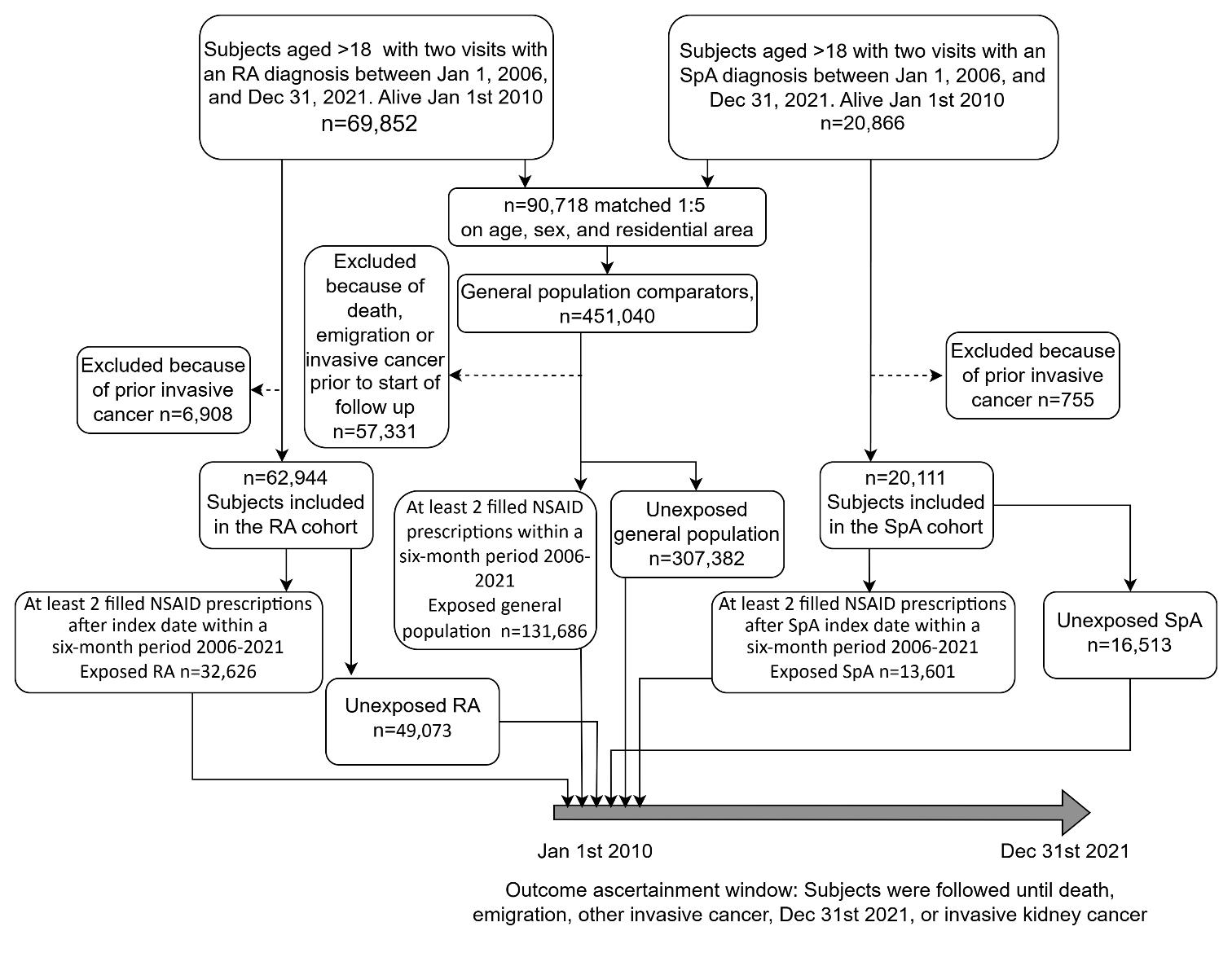


## **Supplementary notes.** NSAID Exposure Classification by Dispensed Prescriptions and DDDs

**Definition of regular use**

To account for regular NSAID use, we required individuals to have at least two dispensed prescriptions recorded in the PDR, with the prescriptions issued within six months of each other. This criterion excluded sporadic or occasional NSAID users who had only one prescription or multiple prescriptions separated by several years. In the regression model start of follow up was here defined as the index date or January 1^st^ 2010, whichever occurred later. Individuals were considered exposed at the date of a second dispensed NSAID within a 6-month period counting from January 1^st^ 2006. For the RA and SpA cohorts, NSAID prescriptions dispensed during the (immortal) time before their index dates (second RA or SpA-diagnosis) were excluded from main analyses.

**Definition of cumulative use**

For cumulative exposure, we converted all dispensed NSAID prescriptions into defined daily doses (DDDs), as outlined by the World Health Organization. Each dispensed prescription contributed to the cumulative total, while unused returned prescriptions were subtracted. In the regression model start of follow up was again defined as the index date or January 1^st^ 2010, whichever occurred later Individuals were considered exposed at the date of a second dispensed NSAID within a 6-month period counting from January 1^st^ 2006. Individuals were counted as exposed < 1 years until their cumulative total reached 366 DDDs, at which point they moved to the 1 to <4 years exposure category. Upon accumulating 1461 DDDs, individuals transitioned to the ≥4 years exposure category. Individuals could only transition to higher exposure categories and were not allowed to revert to lower categories. For the RA and SpA cohorts, NSAID prescriptions dispensed during the (immortal) time before their index dates (second RA or SpA-diagnosis) were excluded from main analyses.

This approach assumed complete adherence to dispensed prescriptions, except for returned unused medications. It also assumed that exposure could be readily translated between NSAIDs according to the DDDs, i.e. that 1.2 g of ibuprofen was equivalent to 0.5 g of naproxen. Finally, the method equated short-term, high-dose NSAID use with the same cumulative DDDs consumed at a lower dose over a prolonged period.

## **Supplementary table 3.** Baseline Covariate Definitions

| **Diabetes mellitus** | |  |
| --- | --- | --- |
| Data Sources | National Patient Register (inpatient/outpatient)  Prescribed Drugs Register |  |
| Definition | Both a prescription of ATC:A10 and any diagnosis using the following ICD-10 codes:  ('E10' 'E11') |  |
| Time Window | - |  |
| **Ischemic heart disease** | |  |
| Data Sources | National Inpatient Register (hospital only) |  |
| Definition | Any diagnosis using the following ICD-10 codes:  ('I20' 'I21' 'I22''I23''I24''I25') |  |
| Time Window | - |  |
| **Congestive Heart Failure** | |  |
| Data Sources | National Patient Register (inpatient/outpatient) |  |
| Definition | Any diagnosis using the following ICD-10 codes:  ('I110' 'I130' 'I132' 'I50') |  |
| Time Window | - |  |
| **Stroke** (Cerebrovascular Disease) | |  |
| Data Sources | National Patient Register (inpatient/outpatient) |  |
| Definition | Any diagnosis using the following ICD-10 codes:  (''I60' 'I61' 'I62' 'I63' 'I64' 'I67' 'I69') |  |
| Time Window | - |  |
| **VTE** (Venous Thromboembolism) | |  |
| Data Sources | National Patient Register (inpatient/outpatient)  Prescribed Drugs Register |  |
| Definition | Any diagnosis using the following ICD-10 codes:  ('I26' 'I80' 'I82' 'O222' 'O223')  Any prescriptions using ATC:  (‘B01AA’ ‘B01AB’ ‘B01AE’ ‘B01AF’ ‘B01AX’)  *Both a diagnosis code and prescription are required for identification. |  |
| Time Window | [-1825 to -1] |  |
| **Chronic kidney failure** | |  |
| Data Sources | National Patient Register (inpatient/outpatient) |  |
| Definition | Any diagnosis using the following ICD-10 codes:  N18 |  |
| Time Window | - |  |
| **Chronic obstructive Pulmonary Disease** | |  |
| Data Sources | National Patient Register (inpatient/outpatient) |  |
| Definition | Any diagnosis using the following ICD-10 codes:  ( 'J43' 'J44') |  |
| Time Window | - |  |
| **Essential hypertension** | |  |
| Data Sources | National Patient Register (inpatient/outpatient) |  |
| Definition | Any diagnosis using the following ICD-10 codes:  (’I10.9’) |  |
| Time Window | - |  |
| **Joint Surgeries** | |  |
| Data Sources | National Patient Register (inpatient) |  |
| Definition | Any of the following procedure codes:  ('NGB' 'NFB' 'NBB' 'NHB' 'NHC' 'NHE' 'NHF' 'NHG' 'NDB' 'NDC' 'NDE' 'NDF' 'NDG') |  |
| Time Window | [-1825 to -1] |  |
| **Hospitalized infections** | |  |
| Data Sources | National Patient Register (inpatient) | |
| Definition | Any diagnosis using the following ICD-10 codes:  (' A00-B99’ 'D73.3', 'E06.0', 'E32.1', 'G04.2', 'H00.0', 'H44.0', 'H60.0', 'H60.1', 'H60.2', 'H60.3' 'I301',  'I40.0', 'J34.0','J38.3', 'J39.0', 'J39.1', 'J44.0', 'K04.4', 'K04.6', 'K04.7', 'K10.2', 'K11.3','K12.2','K14.0',  'K57.0', 'K57.2', 'K57.4', 'K57.8', 'K63.0', 'K65.0', ’K65.1', 'K65.2', 'K65.9', 'L30.3', 'M46.2', 'M46.3',  'M46.4', 'M64.5','M60.0', 'M65.0', 'M71.0', 'M71.1', 'M72.6', 'N15.9', 'N41.2', 'N43.1', 'N45.2',  'N45.3', 'N45.4', 'N48.2', 'N75.1' 'G00', 'G01', 'G02', 'H66', 'H67', 'H70', 'J32', 'J36', 'J85', 'J86', 'K61',  'M00', 'M01', 'M86', 'N12', 'N61', 'N70', 'N73', ' L00-L08’, ' J00-J22’, ' G05-G07’, | |
| Time Window | [-1825 to -1] | |
| **Hospitalized urinary infections** | |  |
| Data Sources | National Patient Register (inpatient) | |
| Definition | Any diagnosis using the following ICD-10 codes:  ('N13.6’ ‘N15.1’ ‘N10’ ‘N11’ ‘N30’ ‘N34.0’ ‘N39.0’) | |
| Time Window | [-1825 to -1] | |
| **Urolithiasis** | |  |
| Data Sources | National Patient Register (inpatient/outpatient) | |
| Definition | Any diagnosis using the following ICD-10 codes:  ('N20' 'N21') | |
| Time Window | - | |
| **csDMARD** prescriptions | |  |
| Data Sources | Prescribed Drugs Register |  |
| Definition | At least two (same or different) dispensed prescription of any csDMARD ('sulfasalazine''Leflunomide''apremilast'‘Azatioprin''guld''guld2''penicillamine'  ‘klorokin''hydroxiklorokin''metotrexat’)- using the following ATC-codes: 'L04AX03''P01BA01''P01BA02''M01CC01''M01CB01''M01CB03'L04AX01' L04AA32' L04AA13' 'A07EC01' |  |
| Time Window | - |  |
| **b/ts-DMARD** prescriptions | |  |
| Data Sources | Prescribed Drugs Register |  |
| Definition | At least two (same or different) dispensed prescription of any b/ts-DMARD-( 'rituximab'‘abatacept''belimumab''etanercept''infliximab'‘adalimumab''certolizumab''golimumab'  'anakinrina''ustekinumab''tocilizumab''secukinumab''ixekizumab''sarilumab'  'tofacitinib''baricitinib''upadacitinib''filgotinib') using the following ATC-codes: ('L01XC02''L04AA24''L04AA26''L04AB01''L04AB02''L04AB04''L04AB05''L04AB06''L04AC03'  'L04AC05''L04AC07''L04AC10''L04AC13''L04AC14''L04AA29''L04AA37''L04AA44''L04AA45') |  |
| Time Window | - |  |
| **AxSPA, RA duration** | |  |
| Data Sources | Swedish Rheumatology Quality Register |  |
| Definition | Number of years between AxSPA or RA debut and cohort entry date. |  |
| Time Window | NA |  |
| **Demographic** Characteristics | |  |
| Data Sources | Total Population Register, LISA |  |
| Definition | Sex, Age (number of years from birth to index treatment start), Level of Education (categorized into: less than twelve years, or twelve years or more). |  |
| Time window | NA |  |
| **Cancer in a full sibling** | |  |
| Data Sources | Cancer register, Multi generation register |  |
| Definition |  |  |
| Time window |  |  |
| **Low dose ASA (75mg/160mg)** | |  |
| Data Sources | Prescribed Drugs Register |  |
| Definition | At least two dispensed prescription of low dose ASA (75mg or 160mg) ATC-code : 'B01AC06) |  |
| Time Window | - |  |
| **Paracetamol (acetaminophen)** | |  |
| Data Sources | Prescribed Drugs Register |  |
| Definition | At least two dispensed prescription of paracetamol ATC-code : 'N02BE01’ |  |
| Time Window | - |  |

## **Supplementary table 4.** Events, crude incidence rates and hazard ratios (HR) of KC in the general population by cumulative years of NSAID exposure measured by DDDs. Alternative categorization of cumulative years of NSAID exposure.

| **NSAID exposure** | **Events n** | **Incidence per 100,000** | **HR (95% CI)** |
| --- | --- | --- | --- |
| Unexposed | 458 | 2,1 | REF |
| < 1 year | 180 | 3,1 | 1.26 (1.05-1.51) |
| 1 to <4 years | 85 | 3,6 | 1.30 (1.06-1.76) |
| 4 to <7 years | 19 | 4,5 | 1.68 (1.05-2.71) |
| 7 to <10 years | 5 | 3,2 | 1.18 (0.48-2.88) |
| >=10 | 4 | 3,3 | - |

## **Supplementary table 5.** Crude incidence rates and hazard ratios (HR) of kidney cancer with NSAID prescriptions dispensed before the index date (second diagnosis of rheumatoid arthritis (RA) or spondyloarthritis (SpA) included.

|  | **NSAID** | **Individuals, n** | **Kidney cancer, n** | **Crude incidence rates /10.000 of kidney cancer** | **HRa (95% CI)*** | **HRb (95% CI)** # |
| --- | --- | --- | --- | --- | --- | --- |
| **RA** | Unexposed | 21,974 | 43 | 3.4 | reference | reference |
|  | Exposed | 46,426 | 98 | 2.8 | 0.94 (0.65-1.35) | 0.91 (0.63-1.31) |
| **SpA** | Unexposed | 4,457 | 7 | 3.1 | reference | reference |
|  | Exposed | 17,601 | 35 | 2.7 | 1.02 (0.45-2.29) | 1.29 (0.56-2.97) |

*Adjusted for calendar year, sex, #Adjusted for calendar year, sex, education, use of paracetamol, use of low dose ASA, KC in a full sibling, comorbidities (CHF, diabetes, Hypertension, COPD, ischaemic heart disease, renal failure, stroke, venous thromboembolism, hospitalized infections total and hospitalized infections, urogenital infections, urolithiasis), rheumatic disease duration, use of glucocorticoids, csDMARDs and bDMARDs at start of follow up.

## **Supplementary table 6.** Hazard ratios (HR) of kidney cancer with ever regular use of NSAID in three cohorts using follow-up time as the time-scale instead of attained age

|  | **HRa (95% CI)*** | **HRb (95% CI)^#^** |
| --- | --- | --- |
| **General population** |  |  |
| NSAID unexposed | reference | reference |
| NSAID exposed | 1.35 (1.16-1.57) | 1.32 (1.13-1.55) |
| **Rheumatoid arthritis** |  |  |
| NSAID unexposed | reference | reference |
| NSAID exposed | 0.94 (0,67-1.33) | 0.89 (0.62-1.29) |
| **Spondyloarthritis** |  |  |
| NSAID unexposed | reference | reference |
| NSAID exposed | 1.22 (0.62-2.41) | 1,64 (0.80-3.38) |

*HRa Adjusted for calendar year, sex, and age (linear and quadratic term) #HRb Adjusted for calendar year, sex, age (linear and quadratic term), education, use of paracetamol, use of low dose ASA, KC in a full sibling, comorbidities (CHF, diabetes, Hypertension, COPD, ischemic heart disease, renal failure, stroke, venous thromboembolism, hospitalized infections total and hospitalized infections, urogenital infections, urolithiasis). RA and AS analyses additionally adjusted for rheumatic disease duration, use of glucocorticoids, csDMARDs and bDMARDs at start of follow up.

## **Supplementary table 7.** Hazard ratios (HR) of kidney cancer with ever regular use of NSAID in three cohorts, excluding the first year of follow-up

|  | **HRa (95% CI)*** | **HRb (95% CI)^#^** |
| --- | --- | --- |
| **General population** |  |  |
| NSAID unexposed | reference | reference |
| NSAID exposed | 1.26 (1.08-1.48) | 1.22 (1.03-1.45) |
| **Rheumatoid arthritis** |  |  |
| NSAID unexposed | reference | reference |
| NSAID exposed | 0.85 (0.60-1.22) | 0.84 (0.56-1,26) |
| **Spondyloarthritis** |  |  |
| NSAID unexposed | reference | reference |
| NSAID exposed | 1.20 (0.61-2.37) | 1.48 (0.70-3.11) |

*HRa Adjusted for calendar year, sex with attained age as the underlying timescale

#HRb Adjusted for calendar year, sex, education, use of paracetamol, use of low dose ASA, KC in a full sibling, comorbidities (CHF, diabetes, Hypertension, COPD, ischemic heart disease, renal failure, stroke, venous thromboembolism, hospitalized infections total and hospitalized infections, urogenital infections, urolithiasis). RA and AS analyses additionally adjusted for rheumatic disease duration, use of glucocorticoids, csDMARDs and bDMARDs at start of follow up. Attained age as the underlying time scale.

## **Supplementary table 8.** TNM cancer stage grouping at diagnosis of KC by regular use of NSAID

|  | **General population, % (n)** | | **Rheumatoid arthritis, % (n)** | | **Spondyloarthritis , % (n)** | |
| --- | --- | --- | --- | --- | --- | --- |
| NSAID | Unexposed | Exposed | Unexposed | Exposed | Unexposed | Exposed |
| Stage 1 | 43 (165) | 42 (106) * | 42 (25) | 45 (25) | 50 (6) | 57 (17) |
| Stage 2 | 10 (39) | 9 (22) | 3 (2) | 16 (9) | 25 (3) | 0 (0) |
| Stage 3 | 17 (66) | 17 (44) | 12 (7) | 11 (6) | 8 (1) | 7 (2) |
| Not Stage 4** | 8 (31) | 6 (14) | 8 (5) | 7 (4) | 8 (1) | 13 (4) |
| Stage 4 | 16 (61) | 21 (54) | 32 (19) | 14 (8) | 8 (1) | 17 (5) |
| Missing | 5 (21) | 5 (12) | 2 (1) | 7 (4) | 0 (0) | 7 (2) |
| Percent | 100 (383) | 100 (252) | 100 (59) | 100 (56) | 100 (12) | 100 (30) |
| Not in the NCR n*** | 75 | 41 | 18 | 8 | 0 | 0 |

TNM= Tumor, lymph node, metastasis according to the National Cancer register, Stage 1=T1 N0 M0 Stage 2=T2 N0 M0 Stage 3= T3 N0 M0 or T 1-3 N1 M0 Stage 4=T4 regardless of N or M, or M1 regardless of T or N.

*Includes one case of T0N0M0

**Lymph node involvement unknown but no distant metastasis and tumor size <T4

*** Cases of KC identified through the causes of death register without any matching observation in the National Cancer Register

## **Supplementary table 9.** Crude incidence rates and hazard ratios (HR) of death due to kidney cancer by ever regular NSAID use

|  | **Individuals, n** | **Kidney cancer, n** | **Crude incidence**  **/10,000 person years** | **HRa (95% CI)*** | **HRc (95% CI****)#** |
| --- | --- | --- | --- | --- | --- |
| **General population** |  |  |  |  |  |
| NSAID unexposed | 375 | 68 | 4.3 | reference | reference |
| NSAID exposed | 249 | 60 | 6.7 | 1.63 (1.14-2.32) | 1.26 (0.87-1.82) |
| **Rheumatoid arthritis** |  |  |  |  |  |
| NSAID unexposed | 58 | 20 | 10.3 | 2.39 (1.43-3.99) | 2.26 (1.32-3.88) |
| NSAID exposed | 52 | 10 | 4.6 | 1.16 (0.59-2.27) | 1.00 (0.50-1.98) |
| **Spondyloarthritis** |  |  |  |  |  |
| NSAID unexposed | 12 | (<5 events) | NA | NA | NA |
| NSAID exposed | 30 | 8 | 7.4 | 1.89 (0.88-4.04) | 3.67 (1.54-8.70) |

*Adjusted for calendar year, sex, # Adjusted for calendar year, sex, and Tumor, lymph node, metastasis (TNM) cancer stage at diagnosis

## **Supplementary table 10.** Crude incidence rates and hazard ratio (HR) of kidney cancer in a subset of patients with rheumatoid arthritis (RA) and spondyloarthritis (SpA) 2012-2021 by ever regular NSAID use, and further adjusted by ever smoking

|  | **Kidney cancer, n** | **Crude incidence**  **/10,000 person years** | **HRa (95% CI)*** | **HRa (95% CI) additionally adjusted for ever smoking** |
| --- | --- | --- | --- | --- |
| **General population** |  |  |  |  |
| NSAID unexposed | 39 | 3.4 | Ref | ref |
| NSAID exposed | 50 | 3.0 | 1.06 (0.69-1.63) | 1.05 (0.69-1.62) |
| **Rheumatoid arthritis** |  |  |  |  |
| NSAID unexposed | 36 | 3.7 | ref | ref |
| NSAID exposed | 37 | 2.9 | 0.89 (0.56-1.41) | 0.88 (0.56-1.41) |
| **Spondyloarthritis** |  |  |  |  |
| NSAID unexposed | 3 | 1.7 | reference | reference |
| NSAID exposed | 13 | 3.3 | NA (<5 events) | NA (<5 events) |

*Adjusted for calendar year and sex and inherently adjusted for age (attained age was time scale)

## **Supplementary table 11.** Crude incidence rate and hazard ratios (HR) of kidney cancer by ever regular use of NSAID restricted to kidney cancer subtype clear cell carcinoma.

|  | **Individuals, n** | **Kidney cancer, n** | **Crude incidence**  **/10,000**  **person years** | **HRa (95% CI)*** | **HRb (95% CI)^#^** |
| --- | --- | --- | --- | --- | --- |
| **General population** |  |  |  |  |  |
| NSAID unexposed | 307,382 | 270 | 1.2 | reference | reference |
| NSAID exposed | 131,686 | 182 | 2.0 | 1,39 (1.15-1.68) | 1.31 (1.07-1.60) |
| **Rheumatoid arthritis** |  |  |  |  |  |
| NSAID unexposed | 49,073 | 42 | 1.9 | 1,22 (0.88-1.69) | 1.09 (0.78-1.53) |
| NSAID exposed | 32,626 | 38 | 1.5 | 1,08 (0.77-1.52) | 0.99 (0.70-1.42) |
| **Spondyloarthritis** |  |  |  |  |  |
| NSAID unexposed | 16,513 | 9 | 1.7 | 2,10 (1.08-4.10) | 1.95 (1.00-3.83) |
| NSAID exposed | 13,601 | 15 | 1.5 | 1.79 (1.06-3.03) | 1.65 (0.97-2.81) |

*Adjusted for calendar year, sex and inherently adjusted for age (attained age was time scale) #Adjusted for calendar year, sex, education, use of paracetamol, use of low dose ASA, KC in a full sibling, comorbidities (CHF, diabetes, Hypertension, COPD, ischemic heart disease, renal failure, stroke, venous thromboembolism, hospitalized infections total and hospitalized infections, urogenital infections, urolithiasis). RA and AS analyses additionally adjusted for rheumatic disease duration, use of glucocorticoids, csDMARDs and bDMARDs at start of follow up. Attained age as the underlying time scale.

## **Supplementary table 12**. Crude incidence rate and hazard ratios (HR) of KC by ever regular NSAID use including only of KC recorded in National Cancer Register

|  | **Individuals, n** | **Kidney cancer, n** | **Crude incidence**  **/10,000**  **person years** | **HRa (95% CI)*** | **HRb (95% CI)^#^** |
| --- | --- | --- | --- | --- | --- |
| **General population** |  |  |  |  |  |
| NSAID unexposed | 307,382 | 383 | 1.8 | reference | reference |
| NSAID exposed | 131,686 | 252 | 2.8 | 1,37 (1.17-1.61) | 1.29 (1.09-1.53) |
| **Rheumatoid arthritis** |  |  |  |  |  |
| NSAID unexposed | 49,073 | 59 | 2.6 | 1,21 (0.92-1.59) | 1.10 (0.83-1.47) |
| NSAID exposed | 32,626 | 56 | 2.3 | 1,13 (0.85-1.50) | 1.06 (0.79-1.42) |
| **Spondyloarthritis** |  |  |  |  |  |
| NSAID unexposed | 16,513 | 12 | 2.3 | 1,99 (1.12-3.54) | 1.84 (1.03-3.29) |
| NSAID exposed | 13,601 | 30 | 3.0 | 2.54 (1.74-3.70) | 2.37 (1.61-3.48) |

*Adjusted for calendar year, sex, #Adjusted for calendar year, sex, education, use of paracetamol, use of low dose ASA, KC in a full sibling, comorbidities (CHF, diabetes, Hypertension, COPD, ischemic heart disease, renal failure, stroke, venous thromboembolism, hospitalized infections total and hospitalized infections, urogenital infections, urolithiasis). RA and AS analyses additionally adjusted for rheumatic disease duration, use of glucocorticoids, csDMARDs and bDMARDs at start of follow up. Attained age as the underlying time scale.
